# Structural basis for redox sensitivity in *Corynebacterium glutamicum* diaminopimelate epimerase: an enzyme involved in l-lysine biosynthesis

**DOI:** 10.1038/srep42318

**Published:** 2017-02-08

**Authors:** Hye-Young Sagong, Kyung-Jin Kim

**Affiliations:** 1 School of Life Sciences, KNU Creative BioResearch Group, Kyungpook National University, Daehak-ro 80, Buk-ku, Daegu 702-701, Korea

## Abstract

Diaminopimelate epimerase (DapF) is one of the crucial enzymes involved in l-lysine biosynthesis, where it converts l,l-diaminopimelate (l,l-DAP) into d,l-DAP. DapF is also considered as an attractive target for the development of antibacterial drugs. Here, we report the crystal structure of DapF from *Corynebacterium glutamicum (Cg*DapF). Structures of *Cg*DapF obtained under both oxidized and reduced conditions reveal that the function of *Cg*DapF is regulated by redox-switch modulation *via* reversible disulfide bond formation between two catalytic cysteine residues. Under oxidized condition, two catalytic cysteine residues form a disulfide bond; these same cysteine residues exist in reduced form under reduced condition. Disulfide bond formation also induces a subsequent structural change in the dynamic catalytic loop at the active site, which results in open/closed conformational change at the active site. We also determined the crystal structure of *Cg*DapF in complex with its product d,l-DAP, and elucidated how the enzyme recognizes its substrate l,l-DAP as a substrate. Moreover, the structure in complex with the d,l-DAP product reveals that *Cg*DapF undergoes a large open/closed domain movement upon substrate binding, resulting in a completely buried active site with the substrate bound.

*Corynebacterium glutamicum* is a Gram-negative bacterium widely utilized for biotechnological production of various amino acids, such as l-glutamate and l-lysine^1^,^2^. As an essential building block for protein synthesis, l-lysine is an industrially important substance for animal feed and dietary supplements. Owing to global increases in meat consumption, l-lysine is particularly required for the animal feed market; the global market for l-lysine is expected to reach USD 6.96 billion by 2020^3^. Furthermore, *meso*-diaminopimelate (d,l-DAP; *m*DAP), the intermediate precursor of l-lysine, is involved in cross-linking of the peptidoglycan cell wall of certain species of Gram-negative bacteria^4^. Because l-lysine biosynthesis is unavailable in animals, members of l-lysine biosynthetic pathway are regarded as attractive targets for antibacterial drug development^5^,^6^.

In *C. glutamicum*, l-lysine is synthesized from aspartate^7^,^8^,^9^. The aspartate is converted into l-aspartate semialdehyde (ASA) by the consecutive reaction of two enzymes. ASA is a precursor for the biosynthesis of various amino acids such as l-threonine, l-isoleucine, l-methionine, and l-lysine. On the l-lysine biosynthetic pathway, ASA is condensed with pyruvate to generate dihydrodipicolinate (DHDP). DHDP reductase reduces DHDP to produce tetrahydrodipicolinate (THDP)^10^. Currently, four different pathways for the biosynthesis of l-lysine that branch out from THDP have been reported in bacteria^11^,^12^: the succinylase pathway, the acetylase pathway, the *m*DAP dehydrogenase pathway, and the recently discovered aminotransferase pathway^13^,^14^. Although most bacteria possess only one of these pathways, *C. glutamicum* utilizes both the succinylase and *m*DAP dehydrogenase pathways^15^. In the succinylase pathway, l,l-diaminopimelate (_L,L_-DAP) is synthesized from THDP by succinylation, transamination, and desuccinylation steps; _L,L_-DAP is converted to d,l-DAP by the action of DAP epimerase (DapF). In contrast, in the *m*DAP dehydrogenase pathway, DAP dehydrogenase converts THDP into d,l-DAP in one step; DAP decarboxylase subsequently catalyzes the decarboxylation of d,l-DAP to form l-lysine.

DapF (EC 5.1.1.7), a pyridoxal 5-phosphate-independent amino acid racemase, catalyzes the stereochemical inversion between l,l-DAP and d,l-DAP. DapF catalyzes the final step of the succinylase pathway. DapF plays an important role in the biosynthesis of l-lysine because DAP decarboxylase only recognizes d,l-DAP. Previous structural and functional studies of DapFs from several organisms such as *Haemophilus influenzae*^16^,^17^,^18^,^19^,^20^,^21^, *Mycobacterium tuberculosis*^22^, *Escherichia coli*^23^, and *Arabidopsis thaliana*^24^ have reported that the DapFs consist of two structurally similar α/β domains and each domain provides one of the two key active site cysteine residues. In addition, DapFs utilizes two cysteine residues for catalysis: one cysteine residue acts as a base and abstracts a proton from _L,L_-DAP, whereas the other acts as an acid and re-protonates the molecule to form d,l-DAP. Despite the importance of *C. glutamicum* in the production of l-lysine, detailed structural and biochemical studies had not been reported on *Cg*DapF prior to this study.

In this study, we determined the crystal structures of *Cg*DapF in both the oxidized and the reduced form. We elucidated that *Cg*DapF is regulated by redox-switch modulation *via* reversible disulfide bond formation between two catalytic cysteine residues, depending on the environmental redox state. We also report that *Cg*DapF undergoes a large open/closed domain movement upon substrate binding.

## Results and Discussion

### Overall structure of *Cg*DapF

**Figure 1:**
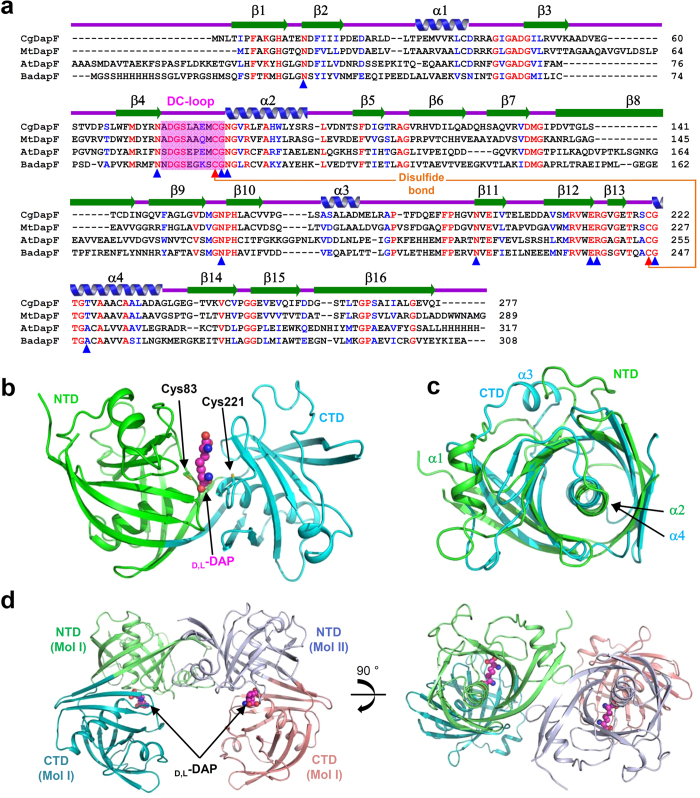
Overall structure of *Cg*DapF. (**a**) Amino acid sequence alignment of the DapF proteins. Identical and highly conserved residues are presented in red and blue colored characters, respectively. Secondary structure elements are shown and labeled based on the structure of *Cg*DapF. The DC-loop is shown in a magenta-colored box. Two catalytic cysteine residues, Cys83 and Cys221, involved in the disulfide bond formation are distinguished with orange-colored line and marked with red colored triangles. Residues involved in the substrate binding are marked with blue colored triangles. Cg, Mt, At, and Ba represent DapF from *Corynebaterium glutamicum, Mycobacterium tuberculosis, Arabidopsis thaliana*, and *Bacillus anthracis*, respectively. (**b**) Monomeric structure of *Cg*DapF. A monomeric *Cg*DapF is shown as a cartoon diagram. NTD and CTD are distinguished with green and cyan colors, respectively. d,l-DAP molecule bound in the enzyme is shown as a sphere model with a magenta color. (**c**) Superimposition of structures of NTD and CTD. The NTD and CTD structure are presented with a color scheme in (**b**). (**d**) Dimeric structure of *Cg*DapF. The dimeric structure of *Cg*DapF is presented as a cartoon diagram with one monomer with a color scheme in (**b**) and the other in light-blue and salmon for the NTD and CTD, respectively. The bound d,l-DAP is shown as a sphere model with a magenta color. The right-side figure is a 90° rotation in the vertical direction from the left-side figure.

To reveal the molecular mechanism of *Cg*DapF, we determined its crystal structure (2.0 Å resolution) *via* the single-wavelength anomalous dispersion method, using a selenium-substituted crystal (Table 1). The structure of *Cg*DapF adopts an overall fold similar to those of diaminopimelate epimerases from *Mycobacterium tuberculosis (Mt*DapF; PDB code 3FVE)^22^, *Arabidopsis thaliana (At*DapF; PDB code 3EJX)^24^, and *Bacillus anthracis (Ba*DapF; PDB code 2OTN) (Fig. 1a). Each *Cg*DapF monomer consists of two distinct domains: an N-terminal domain (NTD; Met1–Asp131 & Gly268–Ile277) and a C-terminal domain (CTD; Met132–Thr267). Each domain contains a set of five-stranded and three-stranded antiparallel β-sheets and two α-helices (Fig. 1b). One α-helix of each domain (α2 in the NTD and α4 in the CTD) is sandwiched between the five-stranded and three-stranded β-sheets, whereas the other helix lies on the surface of the protein. The NTD and the CTD are structurally homologous to each other, with a root-mean-square deviation (RMSD) of 3.4 Å. However, there is one striking structural difference between the two domains; namely, the position of the α-helix that lies on the surface (Fig. 1c). Two catalytic cysteine residues are positioned at the active site, which is located at a cleft between the two domains (Fig. 1b). *Cg*DapF functions as a dimer and the asymmetric unit contains a *Cg*DapF dimer (Fig. 1d), which is consistent with our size-exclusion chromatography results under both the oxidized and the reduced conditions (data not shown). The dimerization interface is mainly constituted by the contacts between β16 from both monomers, and contact between two β-strands connects the two β-sheets of the NTDs from both monomers (Fig. 1d). Contacts between the connecting loops (α1–β3) from both monomers also mediate dimerization of the protein (Fig. 1d). The *PISA*^25^ server was used to calculate the buried interface area (854.9 Å^2^) and the percentage of participating residues (8.3%).

### Reversible disulfide bond formation at the active site of *Cg*DapF

**Figure 2:**
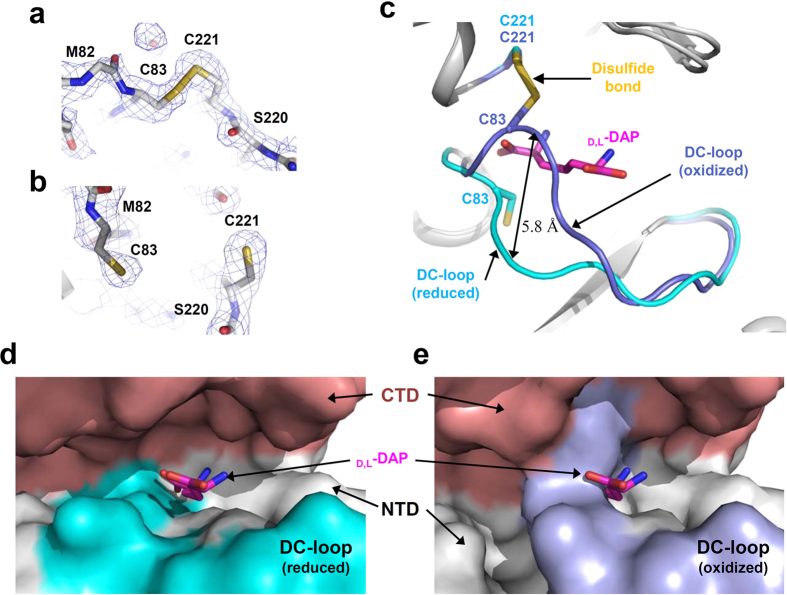
Reversible disulfide bond formation of *Cg*DapF. (**a**,**b**) Electron density map of the two cysteine residues in the oxidized (**a**) and the reduced (**b**) condition. The 2Fo-Fc electron density map of the reversible disulfide bond in the oxidized form and a breakage of the disulfide bond in the reduced form of *Cg*DapF are shown as a blue mesh and contoured at 1.2 σ. (**c**) Disulfide bond-mediated conformational change of DC-loop. The DC-loop in the oxidized form and the reduced form is presented as a cartoon diagram with blue and cyan colors, respectively. Two catalytic cysteine residues involved in the disulfide bond formation are shown as a stick model. The bound d,l-DAP is shown as a stick model with a magenta color. (**d**,**e**) A surface model of the active site of *Cg*DapF. The *Cg*DapF structures in reduced and oxidized form are presented with a surface model with colors of white and salmon for the NTD and CTD, respectively. DC-loops in reduced and oxidized form are shown with the same color scheme as in (**c**).

Although both monomers adopt an almost identical overall shape with respect to each other (RMSD = 0.31 Å), an interesting structural difference was observed between the active sites of each monomer. In one monomer, two catalytic cysteine residues (Cys83 and Cys221) were oxidized, forming a disulfide bond with each other; in contrast, these residues were reduced in the other monomer (Fig. 2a,b). The formation of this disulfide bond also caused a large conformational change in the loop that contains Cys83 (Ala75–Gly84) (Fig. 2c). In the reduced form, the active site exhibits an open conformation that is freely accessible to the substrate (Fig. 2d). In contrast, in the oxidized form of *Cg*DapF, the loop was positioned 5.8 Å closer to the substrate binding site, compared to the reduced form (Fig. 2c), resulting in closure of the substrate binding site (Fig. 2e). Based on these observations, we speculate that the activity of *Cg*DapF is regulated by the environmental redox potential *via* the formation of a reversible disulfide bond and by the subsequent open/closed conformational change at the active site. Because the loop conformation seems to influence the activity of *Cg*DapF directly, we will henceforth refer to this loop as a ‘dynamic catalytic loop’ (DC-loop).

**Figure 3:**
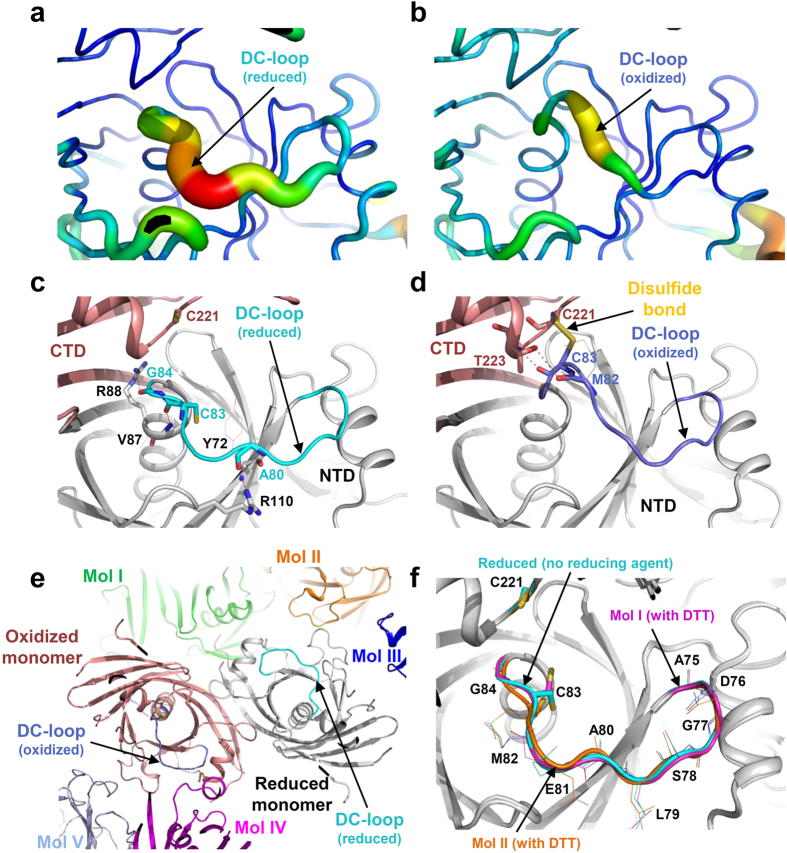
Disulfide bond-mediated conformational change in *Cg*DapF. (**a**,**b**) B-factor presentation of *Cg*DapF structures in the reduced (**a**) and oxidized (**b**) form. Structures in (**a**) and (**b**) are drawn in a same orientation. (**c**,**d**) Stabilization mode of the DC-loop in the reduced (**c**) and oxidized (**d**) form. The DC-loops in the reduced and oxidized form are presented as cartoon diagrams with cyan and blue colors, respectively. Side-chains involved in the stabilization of the DC-loop are shown as stick models. Main-chains involved in the stabilization of the DC-loop are shown as stick models and their side-chains are as line models. Hydrogen bonds are presented as red-colored dotted lines. Structures in (**c**) and (**d**) are drawn in a same orientation. (**e**) Different contacts of *Cg*DapF monomer with the neighboring molecules in the crystal. Oxidized or reduced monomers are shown as a cartoon diagram with salmon and white color, respectively. The DC-loops are distinguished with the same color scheme in (**a**,**b**). The molecules near each monomer are shown as cartoon models with different colors. (**f**) Superposition of reduced monomers in *Cg*DapF. Monomers are shown as a cartoon diagram and distinguished by the color of DC-loop. DC-loop in reduced monomer without reducing agent is colored with cyan. DC-loops from the reduced monomer I and II with DTT are presented as magenta and orange colors, respectively. The catalytic cysteine residues and other residues in DC-loop are presented as a stick and line model, respectively.

The conformational change of the DC-loop seems to result from the high flexibility of the loop. The DC-loop shows much higher *B*-factors in both the oxidized and the reduced forms, as compared with the rest of the protein, indicating that the DC-loop region is highly flexible (Fig. 3a,b). The stabilization modes of the loop are also quite different from each other. In the reduced form of *Cg*DapF, the loop is mainly stabilized by interactions with the NTD. The main chain portion of Ala80 forms direct hydrogen bonds with the main chain portion of Tyr72 and the side chain of Arg110, and the main chain oxygen atoms of Cys83 and Gly84 formed hydrogen bonds with the main chain nitrogen atom of Val87 and the side chain of Arg88 (Fig. 3c). In contrast, in the oxidized form of *Cg*DapF, the DC-loop interacts with the CTD instead. The disulfide bond between Cys83 and Cys221 is the main contributor to the stability of the loop, and the side chain of Thr223 forms hydrogen bonds with the main chain portions of Met82 and Cys83 (Fig. 3d).

In fact, no reducing agent was added, neither to the reservoir nor to the protein solution used for crystallization, indicating that the protein was crystallized under somewhat oxidized condition. Consequently, we sought to answer why each monomer adopted a different conformation and why only one monomer contained a disulfide bond under identical redox condition. When we generated *I*222 symmetry operations, it became clear that the contacts of the two monomers with neighboring molecules completely differ from each other. In particular, the DC-loop of the oxidized monomer makes a close contact with one neighboring molecule, whereas that of the reduced monomer makes no contact with any molecule (Fig. 3e). These observations suggested the possibility that disulfide bond formation at the active site may have been an artifact caused by different crystal contacts, rather that constituting an authentic structural feature of the protein. We then determined a crystal structure of *Cg*DapF in the presence of 1 mM DTT, to investigate whether reversible disulfide bond formation and the associated conformational change we had observed indeed constitute authentic structural features of the protein (Table 1). The structure determined in the presence of 1 mM DTT belonged to space group *I*222, the same group as that of the oxidized structure, and thus identical crystal contacts were formed as when the protein had been crystallized without a reducing agent. Interestingly, under reduced condition the structures of both monomers adopted the same conformation that had been adopted in the ‘reduced monomer’ in the earlier structure that had been determined in the absence of a reducing agent: no disulfide bond was observed in either monomer and the DC-loops showed open conformations (Fig. 3f). These results demonstrate that, in the earlier structure that was determined in the absence of a reducing agent, the pattern of crystal contacts was not responsible for the formation of disulfide bond and the closed conformation. Rather, these features were derived from somewhat oxidized condition during crystallization. Taken together, we propose that *Cg*DapF regulates its function by redox-switch modulation *via* reversible disulfide bond formation and the subsequent open/closed conformational change.

### The redox sensitivity of *Cg*DapF

**Figure 4:**
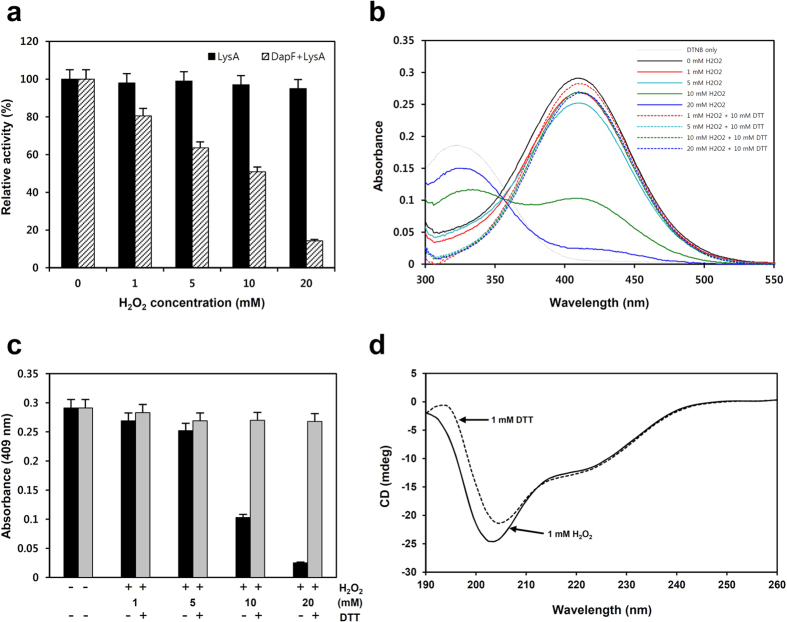
Redox sensitivity of *Cg*DapF. (**a**) Susceptibility of *Cg*DapF to hydrogen peroxide (H_2_O_2_). The *Cg*DapF protein was treated with various concentration of H_2_O_2_ and DapF activity was measured. To test the effect of H_2_O_2_ on LysA, LysA activity with various concentrations of H_2_O_2_ were also measured. (**b**) UV/Vis spectra of *Cg*DapF depending on redox state. The filled-lines indicate the UV/Vis spectra of *Cg*DapF with various concentration of H_2_O_2_, and the dotted-lines indicate those after switch of the redox states to the reduced condition by addition of 10 mM DTT. (**c**) Absorbance at 409 nm of *Cg*DapF depending on redox state. The absorbance values at 409 nm from the Uv/vis spectra of (**b**) are compared. (**d**) The far-UV CD spectra of *Cg*DapF in different redox conditions. Structural changes of *Cg*DapF depending on different redox conditions are measured using the far-UV CD spectra. For the reduced and the oxidized condition, 1 mM each of DTT and H_2_O_2_ are added to the *Cg*DapF protein, respectively.

In general, redox-mediated modification of cellular proteins confers a respose to changes in the environmental redox potential^26^,^27^,^28^. Our crystal structures of *Cg*DapF in both oxidized and reduced forms suggest that *Cg*DapF is regulated by redox-switch modulation, *via* reversible disulfide bond formation. To investigate the redox sensitivity of *Cg*DapF, we examined the susceptibility of *Cg*DapF to hydrogen peroxide (H_2_O_2_). When *Cg*DapF was treated with various concentrations of H_2_O_2_, the enzyme showed a concentration-dependent loss of activity (Fig. 4a), indicating the enzyme is indeed sensitive to oxidative environment. To verify whether disulfide bond formation was indeed reversible, we performed an activity recovery test on *Cg*DapF using an Ellman assay, which measures the amount of free thiol groups in a protein. *Cg*DapF was pre-incubated with various concentrations of H_2_O_2_, and the concentration of free thiol groups in the enzyme was incrementally decreased (Fig. 4b,c). Then, when the environment was switched to reduced condition by removing H_2_O_2_ and adding 10 mM DTT; the oxidized protein indeed recovered and free thiol groups were measured (Fig. 4b,c). In highly oxidized condition, cysteine thiol groups can form sulfinic or sulfonic acids, which cannot be reduced back to thiol groups by DTT. However, the recovery of thiol groups in our Ellman assay results indicate that *Cg*DapF formed disulfide bonds when oxidized by H_2_O_2_ and that the thiol groups of the enzyme were recovered through breakage of disulfide bonds by DTT. Our proposal of a reversible disulfide bond formation that accompanies the structural change was investigated by circular dichroism spectroscopy (CD). CD measurements were recorded over the wavelengths 190–260 nm on the *Cg*DapF protein, which had been treated with either 1 mM H_2_O_2_ or 1 mM DTT (Fig. 4d). The CD results demonstrate that there are notable differences in the ellipticity values recorded from *Cg*DapF that depending on whether it had been treated with DTT or H_2_O_2_, indicating that *Cg*DapF undergoes substantial redox-dependent structural changes. The secondary structure elements calculated using the CD data were quite similar to the structural analysis from the crystal structures, and there was no significant difference between the oxidized and the reduced conditions. Taking our structural and biochemical observations on *Cg*DapF together, we propose that the activity of *Cg*DapF is regulated by redox-switch modulation *via* the reversible disulfide bond formation in response to environmental redox changes. We further propose that the open/closed conformational change caused by the reversible disulfide bond formation also contributes to the redox sensitive regulation of the protein.

### Comparison of *Cg*DapF with other DapFs

The structures of DapF from several organisms have been determined in both oxidized and reduced conditions. To investigate the differences between DapFs, we compared the oxidized form of *Cg*DapF with *Mt*DapF and DapF from *Haemophilus influenzae (Hi*DapF; PDB code 1BWZ and 2Q9H)^16^,^21^ and the reduced form of *Cg*DapF is compared with *Ba*DapF and DapF from *Escherichia coli (Ec*DapF; PDB code 4IJZ)^23^. The overall structure is almost conserved in DapFs and the quaternary structure of DapFs is dimer. In both oxidized and reduced forms of *Cg*DapF, *PISA*^25^ server compute that the oligomeric status is dimer and other DapFs show same results. The dimeric interface of DapFs is also conserved. The two β-strands from both monomers mainly contribute to the dimerization of DapFs. The buried interface areas of *Mt*DapF, *Hi*DapF, *Ba*DapF and *Ec*DapF were calculated by *PISA*^25^ server and the values are 687.7 Å, 1084.6 Å, 854.6 Å, and 771.8 Å, respectively. Similarities in the overall structure extend to the active site residues. In DapF structures, the residues involved in the substrate binding are almost identical. Although Thr223 in *Cg*DapF that stabilizes the carboxyl group of d,l-DAP on the side bearing the d-amino moiety was substituted for Ser219 in *Hi*DapF and *Ec*DapF, the role of the residues seems to be similar each other.

**Figure 5:**
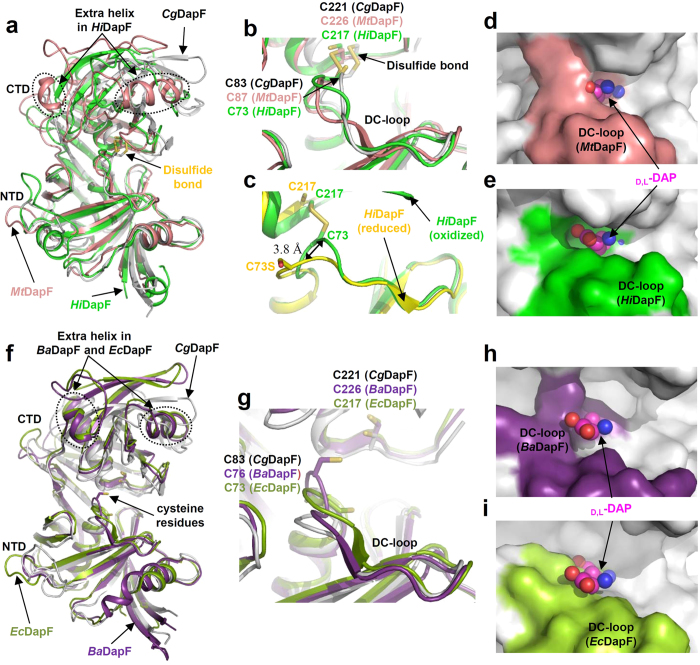
Structural comparison of *Cg*DapF with other DapFs. (**a**) Superimposition of the oxidized form of DapF structures. Structures of the oxidized forms of *Cg*DapF, *Mt*DapF, and *Hi*DapF are superimposed and shown as a cartoon diagram. *Cg*DapF, *Mt*DapF, and *Hi*DapF are distinguished with different colors of gray, salmon, and green, respectively. The disulfide bond are presented as a stick model and labeled. (**b**) Comparison of the DC-loops of DapFs. Structures of *Cg*DapF, *Mt*DapF, and *Hi*DapF are superposed and shown with a color scheme same in (**a**). (**c**) Disulfide bond-mediated conformational change of DC-loop of *Hi*DapF. The DC-loops in the oxidized form and the reduced form are presented as a cartoon diagram with green and yellow colors, respectively. Two catalytic cysteine residues involved in the disulfide bond formation are shown as a stick model. (**d**,**e**) A surface model of the active site of *Mt*DapF and *Hi*DapF. The *Mt*DapF (**d**) and *Hi*DapF (**e**) structures in oxidized form are presented with a surface model. The DC-loops in *Mt*DapF and *Hi*DapF are distinguished with colors of salmon and green, respectively. (**f**) Superimposition of the reduced form of DapF structures. Structures of the reduced form of *Cg*DapF, *Ba*DapF, and *Ec*DapF are superimposed and shown as a cartoon diagram. *Cg*DapF, *Ba*DapF, and *Ec*DapF are distinguished with different colors of gray, purple, and lime green, respectively. The catalytic cysteine residues are presented as a stick model and labeled. (**g**) Comparison of the DC-loop of DapFs. Structures of *Cg*DapF, *Ba*DapF, and *Ec*DapF are superposed and shown with a color scheme same in (**f**). (**h**,**i**) A surface model of the active site of *Ba*DapF and *Ec*DapF. The *Ba*DapF (**h**) and *Ec*DapF (**i**) structures in reduced form are presented with a surface model. DC-loops in *Ba*DapF and *Ec*DapF are distinguished with colors of purple and lime green, respectively.

When we superimposed the NTD of the oxidized form of *Cg*DapF with those of *Mt*DapF and *Hi*DapF, NTD structures matched each other closely, whereas the CTDs from three oxidized DapFs show somewhat different conformations (Fig. 5a). *Hi*DapF contains two additional helices; one helix is inserted at the entrance of CTD and the other is inserted into the connecting loop between β10 and β11 (Fig. 5a). The catalytic cysteine residues from *Cg*DapF and *Hi*DapF form a disulfide bond, whereas those from *Mt*DapF exist in mixed states of the oxidized and the reduced conformations (Fig. 5b). As we mentioned above, the disulfide bond between two catalytic cysteine residues results in the conformational change in the DC-loop. Although the disulfide bond mediated conformational change is also observed in *Hi*DapF, the movement of the DC-loop is less dynamic compared with *Cg*DapF; the loop containing Cys73 (Val70-Gly76) in *Hi*DapF was moved only by 3.8 Å (Fig. 5c). The DC-loop of *Mt*DapF (Asn78-Gly90) is also located at the similar position as that of *Hi*DapF (Fig. 5b). The oxidized conformations also exhibit the closed conformation at the substrate binding site in both *Mt*DapF and *Hi*DapF (Fig. 5d,e). When we compared the reduced form of *Cg*DapF with *Ba*DapF and *Ec*DapF, the conformations of both NTD and CTD are almost identical (Fig. 5e). Compared to the reduced form of *Cg*DapF, *Ba*DapF and *Ec*DapF also contain two additional helices at the similar position to *Hi*DapF (Fig. 5f). The catalytic cysteine residues are all reduced (Fig. 5g) and the DC-loops show the open conformation (Fig. 5h,i). Based on these structural comparisons, we suggest that the activities of DapF proteins might be regulated by redox-switch modulation *via* the reversible disulfide bond formation to respond the environmental redox changes. We also measured the kinetic parameters of *Cg*DapF and the *K*m and kcat values of *Cg*DapF were 1.86 mM and 58 sec-1, respectively. Comparing with the kinetic parameters of other DapFs^29^,^30^, the enzyme activity of *Cg*DapF seems to be somewhat lower than other DapFs.

### Substrate binding mode of *Cg*DapF

**Figure 6:**
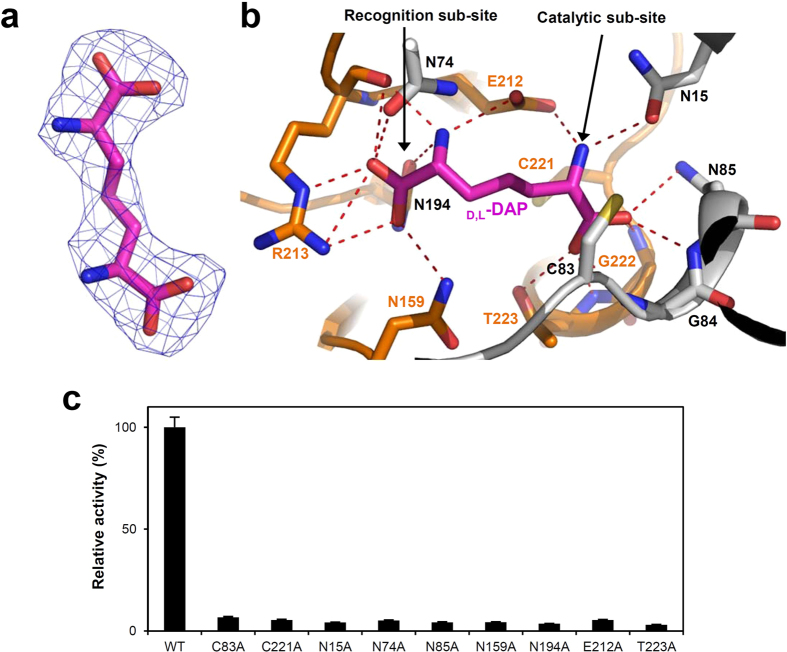
Substrate binding mode of *Cg*DapF. (**a**) Electron density map of d,l-DAP. The Fo-Fc electron density map of the bound d,l-DAP is shown as a blue mesh and contoured at 3.5 σ. (**b**) Substrate binding mode of *Cg*DapF. The bound d,l-DAP is presented in a stick model with a magenta color and labeled. NTD and CTD are distinguished by white and orange colors. Residues involved in the d,l-DAP stabilization are shown as stick models. Hydrogen bonds formed between d,l-DAP and neighboring residues are presented with red dotted lines. (**c**) Site directed mutagenesis of *Cg*DapF. Residues involved in substrate binding are replaced by alanine residues. The relative activity of recombinant mutant proteins are measured and compared with that of wild-type *Cg*DapF. Each experiment was performed in triplicate.

To investigate the substrate binding mode of *Cg*DapF, we determined the crystal structure of the protein in complex with the d,l-DAP product (Table 1). In fact, although we added a mixture of the three DAP enantiomers (l,l-DAP, d,d-DAP, d,l-DAP) into the crystallization solution, we only observed density consistent with d,l-DAP in the electron density map (Fig. 6a). We suspect that the l,l-DAP substrate was converted to the d,l-DAP product during the crystallization procedure because we used the wild-type protein. The substrate binding site is located at a deep cleft formed between the two domains. It can be divided into two sub-sites, a catalytic sub-site and a recognition sub-site, where the d- and l-amino moieties of d,l-DAP are positioned, respectively. The two catalytic cysteine residues are located at the catalytic sub-site, and the amino group in the d-amino moiety is hydrogen bonded to the carboxyl group of Glu212 (Fig. 6b). The carboxyl group of d,l-DAP on the side bearing the d-amino moiety, is stabilized through hydrogen bonds with the main chain nitrogen atoms of Gly84, Asn85, Gly222, and Thr223, and the side chains of Asn85 and Thr223 (Fig. 6b). In the recognition sub-site, the carboxyl group of d,l-DAP on the side bearing the l-amino moiety is stabilized by direct interactions with the main chain of Arg213 and the side chains of Asn74, Asn159, Asn194, and Arg213 (Fig. 6b). The l-amino group of d,l-DAP forms hydrogen bonds with the main chain oxygen atom of Arg213 and side chain of Glu212 (Fig. 6b). In our structure, the stabilization mode of l-amino moiety of d,l-DAP provides a clear explanation of the means by which the enzyme utilizes l,l-DAP as a substrate.

Based on our *Cg*DapF structure in complex with the d,l-DAP product, we performed site-directed mutagenesis experiments to verify residues inferred to be involved in catalysis and substrate binding. We compared the enzyme activities of the resulting mutants to the activity of the wild-type protein. To test their importance as catalytic residues, Cys83 and Cys221 were mutated to alanine; the C83A and C221A mutants almost completely lost their activity (Fig. 6c). This suggests that *Cg*DapF uses both of these residues for catalysis, and the enzymatic reaction mechanism is similar to that of other DapF proteins. In addition, we mutated residues structurally inferred to be involved in the stabilization of the substrate to alanine, generating N15A, N74A, N85A, N159A, N194A, E212A, R213A, and T223A. As anticipated, the enzymatic activities of all these mutants were almost completely abolished, as compared with that of the wild-type enzyme (Fig. 6c).

### Domain movement in *Cg*DapF

**Figure 7:**
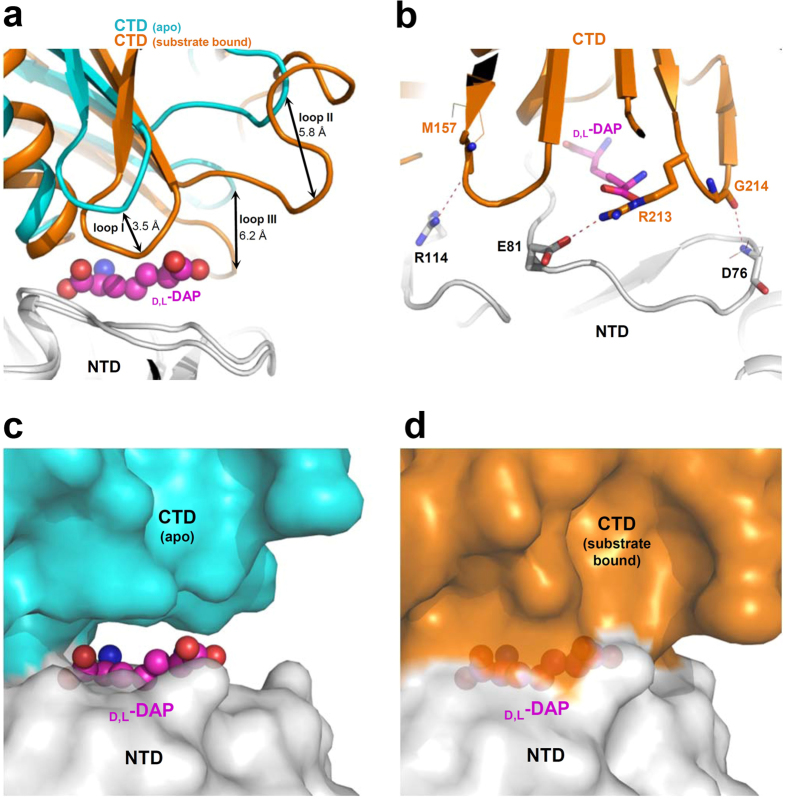
Domain movement of *Cg*DapF. (**a**) Domain movement of *Cg*DapF. *Cg*DapF structure in the apo-form and in complex with _D,L_-DAP are superimposed in a cartoon diagram. In the apo-form, the NTD is shown in white and CTD in cyan colors. In the complex structure, NTD is also presented in a white and CTD in an orange color. The d,l-DAP product is shown as a sphere in a magenta color. Three loops and the distances between structures are labeled. (**b**) Stabilization mode of the reorganized CTD in *Cg*DapF in complex with d,l-DAP. *Cg*DapF is shown as a cartoon diagram with an orange color. Residues involved in the inter-domain interactions are presented as a stick model and labeled. Side-chains involved in the stabilization of the reorganized CTD are shown as stick models. Main-chains involved in the stabilization the reorganized CTD are shown as stick models and their side-chains are as line models. Hydrogen bonds are presented as red-colored dotted lines. (**c**,**d**) Active site closure of *Cg*DapF. *Cg*DapF structures in the apo-form and in complex with d,l-DAP are presented with a surface model with same color scheme in (**a**).

When we superimposed the *apo*-structure of *Cg*DapF with that of the complex with d,l-DAP, it became evident that substrate binding had induced the CTD to move towards the NTD (Fig. 7a). In particular, three loops (Loop I: Met157–Asn159; Loop II: Met177–Val193; Loop III: Arg213–Gly214) move by 3.5 Å, 5.8 Å, and 6.2 Å, respectively (Fig. 7a). For the stabilization of reorganized CTD, residues from NTD interact with the CTD through hydrogen bonds: Met157 from Loop I, and Arg213 and Gly214 from Loop III interact with the Arg114, Glu71 and Asp76 of the NTD, respectively (Fig. 7b). In addition, the interactions between the reorganized CTD and the residues located at the entrance of the active site cleft result in a completely buried substrate binding site (Fig. 7c,d). These structural observations clearly show that *Cg*DapF undergoes an open/closed conformational change during the entrance of the substrate and the release of the product.

In this report, we have revealed that the function of *Cg*DapF is regulated by redox-switch modulation *via* the reversible disulfide bond formation in the response to the environmental redox conditions. This reversible disulfide bond formation also induces structural change in the DC-loop of the active site. Under oxidized condition, the position of the DC-loop occludes the active site, which inactivates the enzyme. However, under reduced condition, the DC-loop undergoes a conformational change that opens the active site, restoring enzymatic activity. Because redox-switch modulation is one of the key regulatory mechanisms used to control the function of enzymes in response to changes in the environmental redox state, we suspect that cellular redox changes might significantly influence l-lysine biosynthesis. Further investigations of the relationship between the cellular redox state and l-lysine biosynthesis in *C. glutamicum* seem to be needed.

## Methods

### Production of *Cg*DapF

The *Cg*DapF gene was amplified by polymerase chain reaction (PCR) using genomic DNA from *C. glutamicum* strain ATCC 13032 as a template. The PCR product was then subcloned into pET30a (Life Science Research), and the resulting expression vector pET30a:*CgdapF* was transformed into the *E. coli* strain B834(DE3), which was grown in 1 L of LB medium containing kanamycin at 37 °C. After induction by the addition of 1 mM isopropyl β-D-1-thiogalactopyranoside (IPTG), the culture medium was maintained for a further 20 h at 18 °C. The culture was then harvested by centrifugation at 4,000 × *g* for 20 min at 4 °C. The cell pellet was resuspended in buffer A (40 mM Tris-HCl, pH 8.0) and then disrupted by ultrasonication. The cell debris was removed by centrifugation at 13,500 × *g* for 30 min and the lysate was applied to an Ni-NTA agarose column (Qiagen). After washing with buffer A containing 30 mM imidazole, the bound proteins were eluted with 300 mM imidazole in buffer A. Finally, trace amounts of contaminants were removed by size-exclusion chromatography by using a Superdex 200 prep-grade column (320 mL, GE Healthcare) equilibrated with buffer A. All purification experiments were performed at 4 °C and SDS-polyacrylamide gel electrophoresis analysis of the purified proteins showed a single polypeptide of 29.9 kDa that corresponded to the estimated molecular weight of the *Cg*DapF monomer. The purified protein was concentrated to 40 mg/mL in 40 mM Tris-HCl, pH 8.0. Site-directed mutagenesis experiments were performed using the Quick Change site-directed mutagenesis kit (Stratagene). The production and purification of the *Cg*DapF mutants were carried out by the same procedure employed for the wild-type protein.

### Crystallization of *Cg*DapF

Crystallization of the purified protein was initially performed with commercially available sparse-matrix screens from Rigaku and Molecular Dimensions by using the hanging-drop vapor-diffusion method at 20 °C. Each experiment consisted of mixing 1.0 μL protein solution (40 mg/ml in 40 mM Tris-HCl, pH 8.0) with 1.0 μL reservoir solution and then equilibrating this against 500 μL reservoir solution. The oxidized form of *Cg*DapF crystals were observed in several crystallization screening conditions. After several optimization steps, the best quality crystals appeared in 1.6 M Ammonium sulfate and 0.1 M Bis-Tris, pH 5.0. The reduced form of *Cg*DapF crystals were crystallized in the condition containing 1.4 M Sodium phosphate monobasic/0.9 M Potassium phosphate dibasic, 0.1 M CAPS, pH 10.0, 0.2 M Lithium sulfate, and 1 mM 1,4-Dithiothreitol (DTT). The crystals of *Cg*DapF in complex with _D,L_-DAP were crystallized in the condition of 1.3 M Sodium citrate and 0.1 M CHES, pH 9.0 and 10 mM DAP isomer.

### Data collection and structure determination of *Cg*DapF

The crystals were transferred to cryoprotectant solution composed of the corresponding conditions described above and 30% (v/v) glycerol, fished out with a loop larger than the crystals, and flash-frozen by immersion in liquid nitrogen. All data were collected at the 7 A beamline of the Pohang Accelerator Laboratory (PAL, Pohang, Korea), using a Quantum 270 CCD detector (ADSC, USA). The oxidized form and reduced form of *Cg*DapF crystals diffracted to a resolution 2.3 and 2.0 Å, respectively. The crystals of *Cg*DapF in complex with _D,L_-DAP diffracted to 2.6 Å resolutions. All data were indexed, integrated, and scaled together using the HKL-2000 software package^31^. The oxidized form of *Cg*DapF belonged to the space group *P*32 with the unit cell parameters *a* = *b* = 143.584 Å, *c* = 122.371 Å, α = β = 90.0°, and γ = 120.0°. In order to solve the phase problem for the crystal, we also prepared crystals from a SeMet derivative. SeMet-substituted oxidized form crystals were obtained using the same crystallization condition as used for the native protein crystal. The crystals of SeMet derivative diffracted to a resolution 2.0 Å and belonged to the space group *I*222 with the unit cell parameters *a* = 101.74 Å, *b* = 119.08 Å, *c* = 155.59 Å, and α = β = γ = 90°. Assuming two molecules of oxidized form of *Cg*DapF (29.2 kDa) per asymmetric unit, the crystal volume per unit of protein mass was approximately 3.94 Å^3^ Da^−1^, which corresponded to a solvent content of 68.80%^32^. The reduced form of *Cg*DapF crystals that substituted with SeMet belonged to the space group *I*222 with unit cell parameters *a* = 101.76, *b* = 118.94 Å, *c* = 153.33 Å, *α* = *β* = *γ* = 90.0°. With two molecules of *Cg*DapF per asymmetric unit, the crystal volume per unit of protein mass was 3.88 Å^3^ Da^−1^, which corresponded to a solvent content of 68.31%^32^. The crystals of *Cg*DapF in complex with _D,L_-DAP belonged to space group *P*4_3_32, with unit cell parameters of parameters *a* = *b* = *c* = 155.7 Å, and *α* = *β* = *γ* = 90.0°. With one molecule of *Cg*DapF in complex with _D,L_-DAP in an asymmetric unit, the crystal volume per unit of protein mass was 2.63 Å^3^ Da^−1^, indicating that the solvent content was approximately 53.26%^32^. The crystal structures of SeMet-substituted *Cg*DapF in oxidized and reduced form were solved by single-wavelength anomalous dispersion (SAD) using data collected at a determined wavelength (0.97934 Å). Experimentally determined *f*” and *f*”’ values by using *SOLVE*/*RESOLVE*^33^. The initial model build was automatically performed using ARP/wARP^34^, and the final model was built by using the program Wincoot^35^. The structure of *Cg*DapF in complex with _D,L_-DAP was determined by molecular replacement with the CCP4 version of MOLREP^36^ using the refined *Cg*DapF in reduced form structure. Model building was performed manually using the program WinCoot^35^, and refinement was performed with CCP4 refmac5^37^. The data statistics are summarized in Table 1. The refined models of oxidized and reduced form of *Cg*DapF and *Cg*DapF in complex with _D,L_-DAP were deposited in the Protein Data Bank with PDB codes of 5H2G, 5H2Y, and 5M47, respectively.

### DapF activity assay

The activity of *Cg*DapF was measured using the coupled enzyme assay with DAP decarboxylase from *C. glutamicum (Cg*LysA). The _D,L_-DAP is generated by the reaction of *Cg*DapF using _L,L_-DAP and then *Cg*LysA immediately converts _D,L_-DAP into _L_-lysine. The production of _l_-lysine was detected using the lysine oxidase/peroxidase method. Lysine oxidase converts the remaining l-lysine into 6-amino-2-oxohexanoate, NH_3_, and H_2_O_2_, and the H_2_O_2_ is then reduced by peroxidase using 2,2′-azino-bis(3-ethylbenzothiazoline-6-sulphonic acid) (ABTS). Immediately after the addition of *Cg*DapF enzyme and *Cg*LysA into the reaction mixture, equal volume of 2x lysine oxidase/peroxidase solution (0.1 unit ml^−1^ lysine oxidase, 1 unit ml^−1^ peroxidase, and 3.6 mM ABTS in 0.1 M potassium phosphate buffer, pH 8.0) was added to the reaction mixture. The amount of oxidized ABTS was detected by measuring absorbance at 412 nm. Activity assays were performed at room temperature and the reaction mixture contains 0.1 M potassium phosphate, pH 8.0, 42.19 μM *Cg*LysA, various concentrations of _L,L_-DAP, and 3.45 μM purified *Cg*DapF enzyme.

To examine the susceptibility of *Cg*DapF to hydrogen peroxide (H_2_O_2_), the *Cg*DapF protein was treated with various concentrations of H_2_O_2_ for 1 h and enzyme-buffer assay mixture was added. To switch the redox condition to the reduced state, the protein solutions treated with various concentrations of H_2_O_2_ were thoroughly dialyzed against the buffer without H_2_O_2_ and 10 mM of DTT was added to the protein samples.

### UV/Vis spectroscopy

To quantify the free thiol group, the protein samples were incubated with 5,5-dithiobis(2-nitrobenzoic acid) (DTNB) and the UV/Vis absorbance spectroscopy was performed. In the presence of thiol group compound, the colorless 5,5-dithiobis(2-nitrobenzoic acid) (DTNB) is converted to yellow 2-Nitro-5-mercaptobenzoic acid (TNB), which shows an absorption maximum at 409 nm. The purified *Cg*DapF protein was pre-incubated with various concentration of H_2_O_2_ for 1 h and then added into the reaction mixture. To switch the environment to reduced condition, 10 mM DTT was added to H_2_O_2_-treated proteins and incubated for 30 min. The reaction mixture contains 0.1 M potassium phosphate, pH 8.0, 10 μM DTNB, and 6.85 μM purified *Cg*DapF enzyme. All incubations and reactions were performed at room temperature.

### Circular Dichroism spectroscopy

CD measurements were carried out with a Jasco J-815 CD Spectropolarimeter. Far-UV CD spectra were collected between 190 and 260 nm with a scan speed 20 nm min^−1^. Spectra of the *Cg*DapF enzymes (33.44 μM in 10 mM potassium phosphate buffer, pH 8.0) reduced by 1 mM DTT or oxidized by 1 mM H_2_O_2_ were recorded at 20 °C in a quartz cuvette (0.2-mm pathlength) with a bandwidth 1 nm. The data were expressed in mean residue ellipticity [*θ*] in degree cm^2^ dmol^−1^.

## Additional Information

**How to cite this article:** Sagong, H.-Y. and Kim, K.-J. Structural basis for redox sensitivity in *Corynebacterium glutamicum* diaminopimelate epimerase: an enzyme involved in l-lysine biosynthesis. *Sci. Rep.* **7**, 42318; doi: 10.1038/srep42318 (2017).

**Publisher's note:** Springer Nature remains neutral with regard to jurisdictional claims in published maps and institutional affiliations.

## Figures and Tables

**Table 1:** Data collection and refinement statistics of *Cg*DapF.

PDB code	*Cg*DapF_Oxidized (SeMet)	*Cg*DapF_Reduced (SeMet)	*Cg*DapF in complex with d,l-DAP
	5H2G	5H2Y	5M47
**Data collection**			
Wavelength (Å)	0.97934	0.97934	0.97934
Cell dimensions (*a, b, c*; *α,β,γ*) (Å; °)	101.7, 119.1, 155.6; 90.0, 90.0 90.0	101.8, 118.9, 153.3; 90.0, 90.0 90.0	155.7 155.7 155.7; 90.0, 90.0, 90.0
Wavelength (Å)	0.97918	0.97924	0.97934
Space group	*I*222	*I*222	*P*4_3_32
Resolution range (Å)	50.00–2.00 (2.03–2.0)	50.00–2.00 (2.03–2.00)	50.00–2.60 (2.64–2.60)
*R*_sym_ or *R*_merge_ (%)	8.2 (40.5)	8.3 (41.4)	11.5 (34.4)
*I*/σ*I*	46.3 (7.4)	22.5 (2.4)	21.1 (4.0)
Completeness (%)	99.8 (100.0)	96.0 (91.8)	97.4 (93.4)
Redundancy	8.1 (8.1)	4.5 (2.7)	12.0 (4.5)
**Refinement**			
Resolution (Å)	50.0–2.0	50.0–2.0	50.0–2.6
No. reflections	59911	58955	19071
*R*_work_/*R*_free_	18.9/21.9	20.7/25.2	19.1/22.9
No. atoms	4563	4273	2154
Protein	4126	4088	2073
Ligand/ion	11	—	13
Water	426	185	68
*B*-factors	37.0	43.0	34.0
Protein	36.0	43.1	34.3
Ligand/ion	37.9	—	30.8
Water	45.8	44.7	27.8
B from Wilson plot (Å^2^)	29.2	28.5	27.6
R.m.s. deviations			
Bond lengths (Å)	0.023	0.022	0.018
Bond angles (°)	2.137	2.180	2.103

^*^Mean B value is for both protein atoms and the solvent molecules.
